# TLR3 Modulates the Response of NK Cells against *Schistosoma japonicum*

**DOI:** 10.1155/2018/7519856

**Published:** 2018-08-30

**Authors:** Jiale Qu, Lu Li, Hongyan Xie, Xiaona Zhang, Quan Yang, Huaina Qiu, Yuanfa Feng, Chenxi Jin, Nuo Dong, Jun Huang

**Affiliations:** ^1^Guangdong Provincial Key Laboratory of Allergy & Clinical Immunology, Sino-French Hoffmann Institute, The Second Affiliated Hospital, Guangzhou Medical University, Guangzhou 511436, China; ^2^Department of Allergy and Clinical Immunology, Guangzhou Institute of Respiratory Diseases, State Key Laboratory of Respiratory Disease, The First Affiliated Hospital, Guangzhou Medical University, Guangzhou 511436, China; ^3^The Sixth Affiliated Hospital of Sun Yat-sen University, Guangzhou 510655, China; ^4^Affiliated Xiamen Eye Center & Eye Institute, Xiamen University, Xiamen 361001, China; ^5^Key Laboratory of Protein Modification and Degradation, School of Basic Medical Sciences, Guangzhou Medical University, Guangzhou 511436, China

## Abstract

Natural killer (NK) cells are classic innate immune cells that play roles in many types of infectious diseases. NK cells possess many kinds of TLRs that allow them to sense and respond to invading pathogens. Our previous study found that NK cells could modulate the immune response induced by *Schistosoma japonicum (S. japonicum)* in C57BL/6 mice. In the present study, the role of TLRs in the progress of *S. japonicum* infection was investigated. Results showed that the expression of TLR3 on NK cells increased significantly after *S. japonicum* infection by using RT-PCR and FACS (*P* < 0.05). TLR3 agonist (Poly I:C) increased IFN-*γ* and IL-4 levels in the supernatant of cultured splenocytes and induced a higher percentage of IFN-*γ*- and IL-4-secreting NK cells from infected mouse splenocytes (*P* < 0.05). Not only the percentages of MHC II-, CD69-, and NKG2A/C/E-expressing cells but also the percentages of IL-4-, IL-5-, and IL-17-producing cells in TLR3^+^ NK cells increased significantly after infection (*P* < 0.05). Moreover, the expression of NKG2A/C/E, NKG2D, MHC II, and CD69 on the surface of splenic NK cells was changed in *S. japonicum*-infected TLR3^−/−^ (TLR3 KO mice, *P* < 0.05); the abilities of NK cells in IL-4, IL-5, and IL-17 secretion were decreased too (*P* < 0.05). These results indicate that TLR3 is the primary molecule which modulates the activation and function of NK cells during the course of *S. japonicum* infection in C57BL/6 mice.

## 1. Introduction

Schistosomiasis japonica is a chronic helminth infection of humans caused by *S. japonicum* [1, 2]. The eggs of *S. japonicum* are deposited in the liver, lung, and intestinal wall and induce granulomatous inflammation and progressive fibrosis, which are the primary clinical pathological changes. There are many types of cells involved in the fight against invading *S. japonicum* and its eggs, including Th cells, natural killer (NK) cells, NKT cells, myeloid-derived suppressor cells (MDSCs), and macrophages [3–6]. Thus, obvious changes could be detected in the immune organs, such as the spleen and local lymph nodes [7, 8].

NK cells are innate lymphocytes that respond rapidly to invading pathogens by exerting a direct cytotoxic effect or secreting various cytokines, particularly interferon-gamma (IFN-*γ*) [7] Recent studies have reported that NK cells are able to survive long enough to take part in the adaptive immune response [9], and NK cells could play an important role in the immune response of host against pathogen and tumor [10]. In parasite infection, both activated and inhibitory receptors such as CD16, CD69, NKG2D, and Ly49a on NK cells were downregulated after a 16-day post-*Angiostrongylus cantonensis* infection in mice [11]. The decrease of circulating frequency of CD56^+^CD161^+^ NK cells in human visceral leishmaniasis [12] and the downmodulation of effector functions in NK cells upon *Toxoplasma gondii* infection were both found too [13]. The negative regulatory role of NK cells in *S. japonicum* egg-induced liver fibrosis was found [14]. Our previous research has found that Th2-like response was induced in the splenic NK cells of *S. japonicum*-infected mice [7].

Toll-like receptors (TLRs) are evolutionarily conserved molecules that were originally identified in vertebrates on the basis of their homology with Toll [15] mammalian TLRs are a family of at least 12 membrane proteins that trigger innate immune responses. The TLR family members are pattern recognition receptors (PRRs) that recognize lipid, carbohydrate, peptide, and nucleic acid structures collectively, which are expressed widely by different groups of microorganisms [16]. TLR2, TLR3, and TLR4 could response to helminth antigens and modulate the activation of dendritic cells during *S. japonicum* infection [17, 18]. TLR3 was reported to modulate immunopathology during *Schistosoma mansoni* egg-driven Th2 responses in the lung [19].

NK cells possess many kinds of TLRs that allow them to sense and respond to invading pathogens. It was reported that in healthy controls, TLR2 and TLR4 of NK cells are mainly intracellular expressed which is similar to TLR9 [20]. TLRs could mediate activation of NK cells in bacterial/viral immune responses in mammals [21]. TLR3 and TLR7 activation in uterine NK cells might play important roles in nonobese diabetic (NOD) mice [22]. Immune response modifiers (IRMs) could modulate NK cell function both in vitro and in vivo, and human NK cell activation was controlled in distinct indirect pathways by TLR7 and TLR8 agonists [23]. In this study, the roles of TLRs on NK cells from the *S. japonicum*-infected mouse spleen were investigated.

## 2. Materials and Methods

### 2.1. Ethics Statement

Six- to eight-week-old female C57BL/6 mice (wild-type, Laboratory Animal Centre of Sun Yat-sen University, China) were used for the experiments. Experiments were also performed by using TLR3^−/−^ mice (B6; 129S1-Tlr3^tm1Fl^v/J) purchased from Model Animal Resource Information Platform (Nanjing, China; strains: J005217). All mice were maintained in a specific pathogen-free microenvironment at the Laboratory Animal Centre, Guangzhou Medical University. Animal experiments were performed in strict accordance with the regulations and guidelines of the institutional animal care and use committee of Guangzhou Medical University. All protocols for animal use were approved to be appropriate and humane by the institutional animal care and use committee of Guangzhou Medical University (2012-11). Every effort was made to minimize suffering.

### 2.2. Mice, Parasites, and Infection

The *Schistosoma japonicum* cercariae used in experiments were obtained from *Oncomelania hupensis-*infected snails (Jiangsu Institute of Parasitic Disease, China). 30 C57BL/6 mice and 10 TLR3^−/−^ mice were percutaneously infected with 40 ± 5 cercariae, and an equal number of uninfected normal mice were used as control. All mice were sacrificed 6 weeks after *S. japonicum* infection as reported before [5].

### 2.3. Antibodies

The following monoclonal antibodies were used for these studies: PE-conjugated rat IgG1 (R3-34), APC-conjugated rat IgG1 (R3-34), APC-cy7-conjugated anti-mouse CD3 (145-2C11), Alexa Fluor 647-conjugated anti-mouse TLR2 (6C2), PE-conjugated anti-mouse TLR4 (MTS510), PE-conjugated anti-mouse TLR7 (A94B10), PerCP-Cy5.5-conjugated anti-mouse CD4 (RM4-5), APC-conjugated anti-mouse CD8 (RPAT8), FITC-conjugated anti-mouse *γδ*TCR (GL3), APC-cy7-conjugated anti-mouse CD11b (M1/70), PE-conjugated anti-mouse Ly6G (1A8), FITC-conjugated anti-mouse CD94 (20d5), PE-conjugated anti-mouse CD314 (XMG1.2), APC-conjugated anti-mouse IFN-*γ* (XMG1.2), PE-conjugated anti-mouse IL-4 (11B11), PE-conjugated anti-mouse IL-17A (TC11-18H10), and APC-conjugated anti-mouse IL-5 (TRFK5). All antibodies were purchased from BD Pharmingen (San Diego, CA, USA). FITC-conjugated rat IgG1 (G0114F7), FITC-conjugated anti-mouse MHC II (M5/114.15.2), FITC-conjugated anti-mouse CD94 (Kp43), PE-cy7-conjugated rat IgG1 (G0114F7), PE-cy7-conjugated anti-mouse F4/80 (EMR1, Ly71), PE-cy5-conjugated anti-mouse CD19 (6D5), PE-cy7-conjugated anti-mouse NK1.1 (PK136), APC-conjugated rat IgG1 (G0114F7), APC-conjugated anti-mouse TLR3 (11F8), PE-conjugated anti-mouse TLR3 (11F8), PE-conjugated anti-mouse NKG2D (A10), and APC-conjugated anti-mouse CD69 (H1.2F3) antibody were purchased from BioLegend (San Diego, CA, USA).

### 2.4. Preparation of Splenocytes and NK Cells

Mice were sacrificed after infection for 6 weeks. The spleens were mechanically dissociated and processed through a 100 *μ*m cell strainer (BD Falcon). After erythrocyte was removed by RBC lysis buffer (NH_4_Cl 8.29 g, KHCO_3_ 1 g, and Na_2_EDTA 37.2 mg per liter), the cells were washed twice in Hanks' balanced salt solution and resuspended in complete RPMI-1640 medium, which contained 10% heat-inactivated fetal calf serum, 100 U/ml penicillin, 100 *μ*g/ml streptomycin, 2 mM glutamine, and 50 *μ*M 2-mercaptoethanol. Splenocytes were counted under microscope and then diluted to a final concentration of 2 × 10^6^ cells/ml for cell culture. For cell staining, splenocytes were stained with fluorescent-labeled anti-mouse CD3 and NK1.1 antibodies for 30 min, followed by washing twice and resuspending in sterile PBS with 0.5 Bull Serum Albumin (BSA). Then, CD3^−^NK1.1^+^ NK cells were sorted by using flow cytometry (MoFlo XDP, Beckman, USA). The purity of NK cells was above 90%, which was used immediately after sorting.

### 2.5. Cell Surface and Intracellular Staining

Splenic lymphocyte stimulation was performed as previously described [7]. Briefly, for cell surface staining, single splenic lymphocyte suspensions were washed twice and stained with anti-CD3, NK1.1, TLR2, TLR4, CD8, CD4, *γδ*T, CD19, Ly6G, F4/80, MHC II, CD69, CD94 (NKG2A/C/E), or CD314 (NKG2D) antibodies for 30 min at 4°C in the dark. Stained cells were washed twice and detected by using flow cytometry. For intracellular cytokine staining, single splenic lymphocyte suspensions were incubated for 5 h in the presence of phorbol 12-myristate 13-acetate (PMA) (20 ng/ml, Sigma) and ionomycin (1 *μ*g/ml, Sigma) at 37°C under a 5% CO_2_ atmosphere; brefeldin A (10 *μ*g/ml, Sigma) was added 1 hour after stimulation. Cells were washed twice and stained with antibodies of cell surface markers for 30 min at 4°C in the dark. Cells were fixed with 4% paraformaldehyde and permeabilized overnight at 4°C in PBS buffers containing 0.1% saponin (Sigma), 1% BSA, and 0.05% NaN_3_. In the next day, cells were stained with conjugated antibodies specific for TLR3, TLR7, IL-4, IFN-*γ*, IL-17, and IL-5 for 30 min. Stained cells were washed twice and detected by using flow cytometry (CytoFLEX, Beckman Coulter, USA), and data were analyzed by the program CytExpert 1.1 (Beckman Coulter, USA).

### 2.6. RNA Preparation for Real-Time PCR

Total RNA of splenic lymphocytes or NK cells was isolated by using TRIzol reagent (Invitrogen Life Technologies, Carlsbad, CA, USA). 1 *μ*g of total RNA was transcribed to cDNA by using a SuperScript III Reverse Transcriptase Kit (Qiagen, Valencia, CA). The following primers were synthesized by Invitrogen (Shanghai, China): for TLR2, 5-CTCTCCGTCCCAACTGATGA-3 (forward) and 5-GGTCTGGTTGCATGGCTTTT-3 (reverse); for TLR3, 5-ATTCGCCCTCCTCTTGAACA-3 (forward) and 5-TCGAGCTGGGTGAGATTTGT-3 (reverse); for TL4, 5-AGGTTGAGAAGTCCCTGCTG-3 (forward) and 5-GGTCCAAGTTGCCGTTTCTT-3 (reverse); for TLR7, 5-GCATTCCCACTAACACCACC-3 (forward) and 5-ACACACATTGGCTTTGGACC-3 (reverse); for *β*-actin, 5-CCGTAAAGACCTCTATGCCAAC-3 (forward) and 5-GGGTGTAAAACGCAGCTCAGTA-3 (reverse). mRNA expression was analyzed with RT-qPCR by using Takara SYBR Premix Ex Taq II (RR820A). PCR conditions were listed as follows: 95°C for 30 s, followed by 40 cycles of 95°C for 5 s and 60°C for 30 s. Amplification was carried out in triplicate. *β*-Actin mRNA was used for normalization. Amplification was performed by using the CFX96 touch qPCR system (Bio-Rad, Hercules, CA, USA), and quantitative mRNA levels were normalized to *β*-actin mRNA expression levels. The levels of TLR transcripts were normalized to *β*-actin transcripts by using the relative quantity (RQ) = 2^−△△Ct^ method. qPCR products were analyzed on a 1.0% multiwelled agarose gel. Electrophoresis was performed in 1× TAE buffer at 400 V for 30 min. The gel was visualized in a ChemiDoc XRS Universal Hood II (Bio-Rad Laboratories).

### 2.7. ELISA Detection

Cells were cultured with different stimulations in 96-well plates at 37°C under a 5% CO_2_ atmosphere for 72 h. Supernatant was collected, and the levels of IFN-*γ* and IL-4 were detected by using ELISA according to the manufacturer's instructions (IFN-*γ*: 551866, BD; IL-4: 555232, BD). The lower detection limit for IFN-*γ* is 3.126 pg/ml and 7.8 pg/ml for IL-4. Samples were read at 450 nm by using a microplate reader (Moder ELX-800, BioTek).

### 2.8. Statistical Analysis

**Figure 1:**
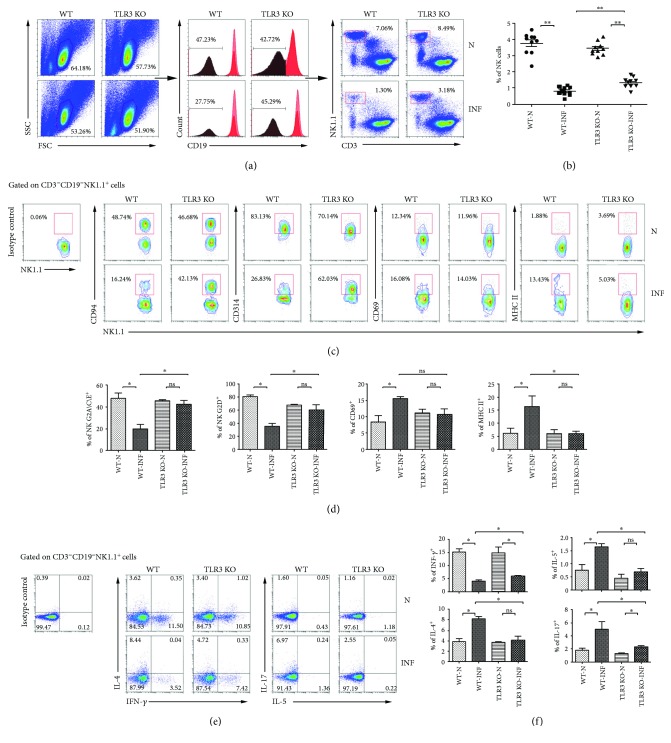
Characteristics of NK cells in *S. japonicum*-infected TLR3^−/−^ mice. Female C57BL/6 wild-type mice (WI) and TLR3^−/−^ mice (TLR3 KO) were infected with *S. japonicum* cercariae or not and sacrificed after 5 weeks. Splenic lymphocytes were isolated and stained by different fluorescent-labeled antibody and analyzed by FACS. (A/B) CD19^−^ cell population was gated firstly. The percentage of CD3^−^NK1.1^+^ NK cells was analyzed. (a) A representative result is shown. (b) Statistical results of the percentage of NK cells in the spleen were calculated from FACS data. One syndrome represents an independent experiment. (c, d) The expression of MHC II, CD69, NKG2A/C/E, and NKG2D on NK cells of mice from both the normal and infected WT and TLR3 KO mouse spleen were detected. (c) A representative result is shown. (d) Statistical results of at least three independent experiments are shown. (e, f) Splenic lymphocytes were stimulated with PMA plus ionomycin for 5 hours, stained by the means of intracellular staining, and analyzed by flow cytometry. The expressions of IFN-*γ*, IL-4, IL-5, and IL-17 on NK cells from different groups are shown. (e) A representative result is shown. (f) The statistical results of at least three independent experiments are shown. Data are shown as mean + SEM of 3–5 samples from three independent experiments. ^∗^*P* < 0.05 and ^∗∗^*P* < 0.01; ns for *P* > 0.05. N: normal; INF: infected.

Data were analyzed with SPSS 11.0 software (SPSS Inc., Chicago, IL, USA). Differences in mean values between groups were assessed by using Mann–Whitney *U* test. One-way ANOVA was used to analyze data of TLR3 KO mouse in Figure 1. *P* < 0.05 was considered statistically significant.

## 3. Results

### 3.1. Increasing TLR3 Expression on Splenic NK Cells in *S. japonicum*-Infected Mice

**Figure 2:**
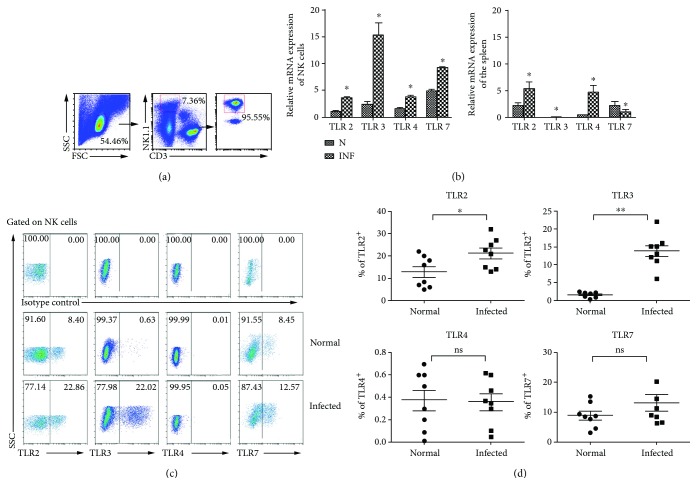
Expression of different TLRs in mouse splenic lymphocytes and NK cells after *S. japonicum* infection. (a) Splenic lymphocytes were separated from normal and *S. japonicum*-infected wild-type mice, and CD3^−^NK1.1^+^ NK cells were isolated from splenic lymphocytes by using flow cytometry and the purity of isolated splenic NK cells was identified by FACS. (b) Total RNA of splenic lymphocytes and NK cells was harvested, respectively. The accumulation of TLR2, TLR3, TLR4, and TLR7 mRNA was quantified by using qPCR. The levels of TLR transcripts were normalized to *β*-actin transcripts by using the relative quantity (RQ) = 2^−△△Ct^ method. Data represent means ± SEM of at least three experiments. (c, d) Expression of TLR2, TLR3, TLR4, and TLR7 on splenic NK cells was assessed by using flow cytometry. (c) A representative result is shown. (d) Statistic results of 6 to 8 independent results are shown. N: normal; INF: infected; ^∗^*P* < 0.05, comparison between the normal and infected groups.

To explore the role of TLRs on NK cells in the progress of *S. japonicum* infection, the expression of TLRs on NK cells from *S. japonicum*-infected mice was detected firstly. As shown in Figure 2, the spleens from normal and infected mice were isolated, and single cell suspensions were prepared, and different fluorescent-labeled anti-mouse CD3 and NK1.1 antibodies were used to sort CD3^−^NK1.1^+^ NK cells by FACS. The purity of sorted NK cells was above 95%. Moreover, mRNA was extracted from both splenocytes and splenic NK cells, respectively. cDNAs were synthesized, and qPCR was performed. The results demonstrated that the expressions of TLR2, TLR3, TLR4, and TLR7 mRNA in splenic NK cells significantly increased after *S. japonicum* infection (*P* < 0.05). TLR3 mRNA expression increased almost fivefold when compared to normal mice (*P* < 0.01). Moreover, splenocytes were stained and the expression of TLR2, TLR3, and TLR4 on CD3^−^NK1.1^+^ NK cells was detected (Figure 2(c)). Results (Figure 2(d)) showed that the percentages of TLR2 and TLR3 in splenic NK cells in the infected group were higher than the normal group (TLR2: 12.80 ± 2.442 versus 21.23 ± 2.409, *P* < 0.05; TLR3: 13.75 ± 1.623% versus 1.558 ± 0.266%, *P* < 0.01). However, there were no significant differences in the expressions of TLR4 and TLR7 on NK cells between normal and infected mice (*P* > 0.05).

### 3.2. Poly I:C Promotes IL-4 Secretion from Infected Mouse Splenic NK Cells

**Figure 3:**
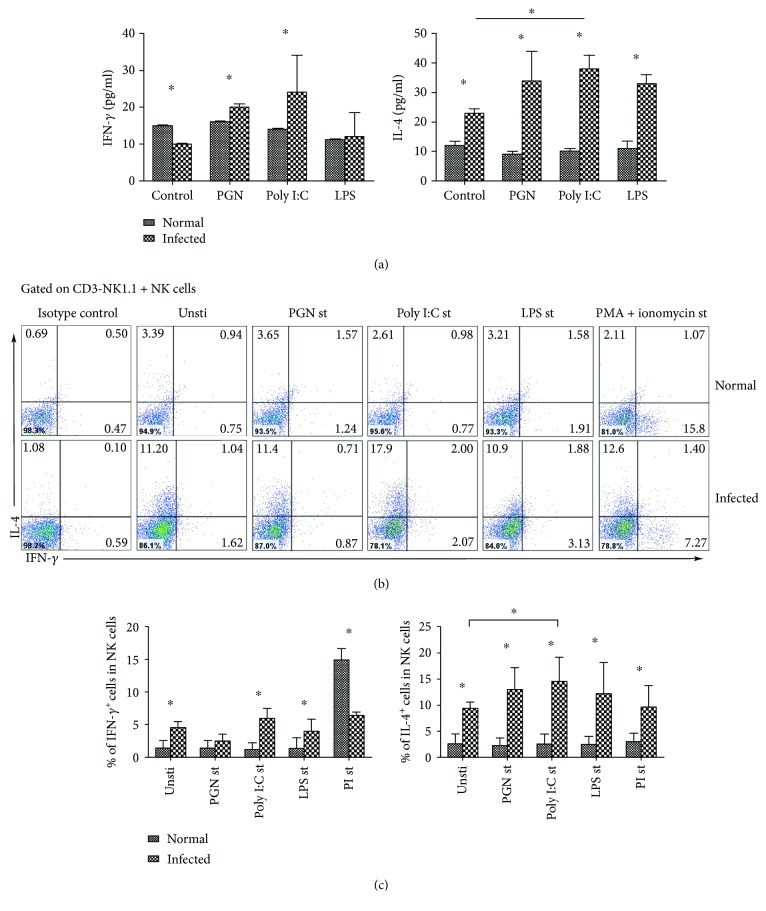
IFN-*γ* and IL-4 secretion induced by TLR agonists. (a) Splenic lymphocyte suspensions from normal and infected mice were prepared and cultured with PGN, Poly I:C, or LPS. Seventy-two hours later, IFN-*γ* or IL-4 production was assessed by using ELISA. (b) Splenic lymphocyte suspensions from normal and infected mice were prepared and stimulated with PGN, Poly I:C, LPS, or PMA plus ionomycin for 5 hours. IFN-*γ* and IL-4 production by splenic NK cells was assessed by using flow cytometry. Results of IFN-*γ* and IL-4 produced by splenic NK cells are calculated and shown (c). Data are shown as mean + SEM of 5 samples from three independent experiments. ^∗^*P* < 0.05, comparison between the normal and infected groups.

To further explore the roles of TLRs on NK cells, splenocytes from normal and infected mice were cultured with PGN, poly I:C, or LPS, respectively. Supernatants were collected 72 h later, and IFN-*γ* and IL-4 levels were detected by using ELISA. As shown in Figure 3(a), the IL-4 level in the supernatants of infected mice was significantly higher than normal mice (*P* < 0.05). After poly I:C stimulation, IL-4 level was significantly higher than the nonstimulated controls (38.76 ± 4.45% versus 23.88 ± 2.33%, *P* < 0.05).

Furthermore, splenocytes from both infected mice and normal mice were stimulated by using PGN, Poly I:C, LPS, or PMA plus ionomycin, respectively. The expression of IFN-*γ* and IL-4 in NK cells was detected via intracellular cytokine staining (Figure 3(b)). As shown in Figure 3(c), the percentage of IL-4^+^ cells in splenic NK cells from infected mice was significantly increased (unst: 2.52 ± 2.00% versus 9.37 ± 1.22%; PGN: 2.32 ± 1.37% versus 13.03 ± 4.11%; Poly I:C: 2.54 ± 1.97% versus 14.60 ± 4.53%; LPS: 2.44 ± 1.67% versus 12.17 ± 6.00%; PI: 3.04 ± 1.55% versus 9.6 ± 4.15%, *P* < 0.05). The percentage of NK^+^IL-4^+^ cells induced by poly I:C was significantly higher than the control group (*P* < 0.05). The percentage of IFN-*γ*^+^ cells in splenic NK cells from infected mice was higher than normal mice (unst: 4.54 ± 0.95%; PGN: 2.42 ± 1.08%; Poly I:C: 6.01 ± 1.50%; LPS: 4.02 ± 1.80%; PI: 6.11 ± 0.47%, *P* < 0.05), but the percentage of IFN-*γ*^+^ NK cells induced by these TLRs was not higher than the unstimulated controls in infected mice (*P* > 0.05).

### 3.3. Cellular Source of TLR3 in the Spleens of *S. japonicum*-Infected Mice

**Figure 4:**
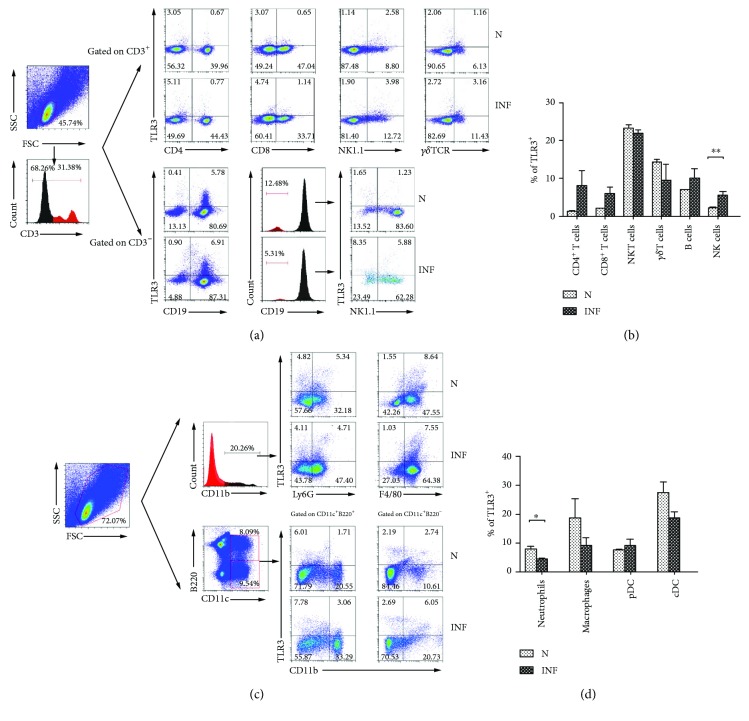
Expression of TLR3 on different spleen cell subsets. (a) Single spleen cell suspensions were separated, the population of small size live cell was gated firstly, and the expression of TLR3 on CD4^+^ T cells, CD8^+^ T cells, NKT cells, and *γδ*T cells in CD3^+^ cell population, CD3^−^CD19^+^ B cells, and CD3^−^CD19^−^NK1.1^+^ NK cells was analyzed by flow cytometry. Statistical analysis of expression of TLR3 on different lymphocyte subsets of the spleen is shown in (b). (c) Splenic lymphocytes were gated on the total live cell population firstly; the expression of TLR3 on different myeloid subsets containing CD11b^+^Ly6G^+^ neutrophils, CD11b^+^F4/80^+^ macrophages, CD11c^+^B220^+^CD11b^−^ plasmacytoid dendritic cells (pDCs), and CD11c^+^B220^−^CD11b^+^ conventional dendritic cells (cDCs) is shown in (d). Data represent means ± SD of three independent experiments with 3–5 mice per group. ^∗∗^*P* < 0.01; ^∗^*P* < 0.05. N: normal; INF: infected.

Single splenocyte suspensions were prepared from normal and *S. japonicum-*infected mice to investigate the cellular source of TLR3 in the *S. japonicum-*infected mouse spleen. TLR3 expression was detected by intracellular staining in different cell subsets, including CD3^+^CD4^+^ Th cells, CD3^+^CD8^+^ Tc cells, CD3^+^NK1.1^+^ NKT cells, CD3^+^*γδ*TCR^+^*γδ*T cells, CD3^−^CD19^+^ B cells, and CD3^−^CD19^−^NK1.1^+^ NK cells in the mononuclear cell population. Figures 4(a) and 4(b) showed that the proportion of TLR3^+^ NK cells was increased approximately threefold after infection (normal: 2.07 ± 0.63%; infected: 5.65 ± 0.78%, *P* < 0.01). There were no significant differences in detection of Th cells, Tc cells, B cells, NKT cells, or *γδ*T cells (*P* > 0.05).

At the same time, TLR3 expression was detected in the myeloid cell populations, including CD11b^+^Ly6G^+^ neutrophils, CD11b^+^F4/80^+^ macrophages, CD11c^+^B220^+^CD11b^−^ plasmacytoid dendritic cells (pDCs), and CD11c^+^B220^−^CD11b^+^ conventional dendritic cells (cDCs). As shown in Figures 4(c) and 4(d), TLR3 was highly expressed on (the surface of?) neutrophils from normal mice (8.03 ± 0.97%), but the proportion declined after *S. japonicum* infection (4.46 ± 0.51%, *P* < 0.05, Figures 4(c) and 4(d)). Similar trend was also found on macrophages and cDCs, but there was no statistical difference (*P* > 0.05). No significant difference between pDCs and cDCs was detected (*P* > 0.05).

### 3.4. Phenotypic and Functional Changes in TLR3^+^ Splenic NK Cells Induced by *S. japonicum*

**Figure 5:**
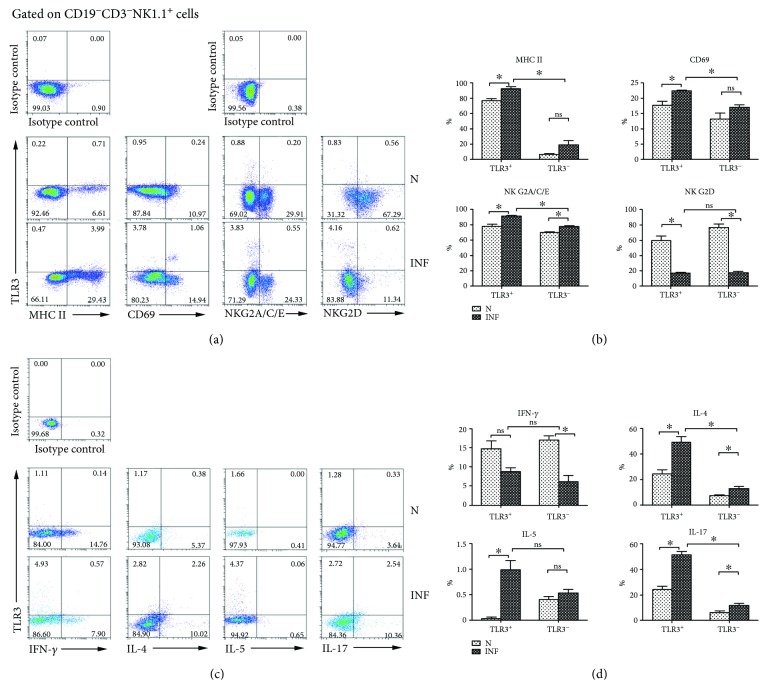
Phenotypic and functional changes in TLR3^+^ splenic NK Cells. (a, b) Splenic lymphocytes were gated on CD19^−^CD3^−^NK1.1^+^ cells firstly, and cells were stained with monoclonal antibodies against mouse NKG2A/C/E, NKG2D, MHC II, and CD69. A representative result is shown in (a). The percentage of activated molecule expression in TLR3^+^ NK cells and TLR3^−^ NK cells was calculated from FACS data (b). (c, d) Splenic lymphocytes were stimulated with PMA plus ionomycin for 5 hours and fixed and stained by using monoclonal antibodies. (c) A representative result is shown. (d) The percentage of cytokine production in TLR3^+^ NK cells and TLR3^−^ NK cells was calculated from FACS data. Data was from three independent experiments with 3–5 mice per group and shown as mean ± SEM. ^∗^*P* < 0.05; ns for *P* > 0.05. N: normal; INF: infected.

Moreover, splenocytes from normal and infected mice were isolated and stained with different fluorescent-labeled antibodies, including anti-CD19, CD3, NK1.1, TLR3, MHC II, CD69, NKG2A/C/E, and NKG2D to investigate the role of *S. japonicum* on TLR3^+^ splenic NK cells. The CD19^−^ mononuclear cells were gated firstly; the expressions of MHC II, CD69, NKG2A/C/E, and NKG2D were detected on TLR3^−/+^ NK cells (Figure 5(a)). The results (Figure 5(b)) demonstrated that the percentages of MHC II-, CD69-, and NKG2A/C/E-expressing cells in TLR3^+^ NK cells significantly increased after infection (MHC II: 76.59 ± 3.054% versus 92.92 ± 1.770%; CD69: 17.72 ± 1.257% versus 22.30 ± 0.3512%; NKG2A/C/E: 77.80 ± 2.498% versus 90.48 ± 1.626%, *P* < 0.05). On the contrary, the percentage of NKG2D on TLR3^+^ NK cells was significantly decreased after infection (47.43 ± 4.974% versus 12.99 ± 1.155%, *P* < 0.05). The expression of NKG2A/C/E on TLR3^−^ NK cells significantly increased after infection (70.23 ± 0.6203% versus 77.18 ± 1.348%, *P* < 0.05), while the percentage of NKG2D on TLR3^−^ NK cells was significantly decreased after infection (61.08 ± 3.683 versus 13.64 ± 1.682, *P* < 0.05). No significant difference was found in the expression of MHC II and CD69 on the surface of TLR3^−^ NK cells (*P* > 0.05).

At the same time, splenocytes from normal and *S. japonicum*-infected mice were isolated and stimulated by PMA plus ionomycin, and intracellular cytokine staining was done as described in Materials and Methods. The expression of IFN-*γ*, IL-4, IL-5, and IL-17 was investigated on TLR3^−^/^+^ splenic NK cells (Figure 5(c)). Results (Figure 5(d)) showed that the percentages of IL-4-, IL-5-, and IL-17-producing cells in TLR3^+^ NK cells population significantly increased after infection (IL-4: 14.73 ± 1.968% versus 8.787.97581%; IL-5: 0.03333 ± 0.03333% versus 0.9833 ± 0.1922%; IL-17: 24.50 ± 2.466% versus 51.76 ± 2.258%, *P* < 0.05). However, no significant difference was found in the percentage of IFN-*γ*-producing cells in TLR3^+^ NK cells (*P* > 0.05). In the population of TLR3^−^ NK cells, the percentages of IL-4- and IL-17-producing cells significantly increased (IL-4: 7.150 ± 1.028% versus 12.85 ± 1.627%; IL-17: 6.223 ± 1.309% versus 11.98 ± 1.534%, *P* < 0.05). However, the percentage of IFN-*γ*-secreting cells significantly decreased (16.96 ± 1.190% versus 6.120 ± 1.609%, *P* < 0.05). No significant difference was found in the percentage of IL-5-producing NK cells (0.4067 ± 0.05812% versus 0.5267 ± 0.08192%, *P* > 0.05).

### 3.5. TLR3 Mediates the Function of Splenic NK Cells during *S. japonicum* Infection

Furthermore, wild-type (WT) and TLR3 knockout (TLR3 KO) mice were infected with *S. japonicum* to confirm the role of TLR3 on NK cells. Splenocytes were isolated 5 weeks after infection, and the noninfected wild-type and TLR3 knockout mice were sacrificed as the control groups. As shown in Figure 1(a), the percentage of CD3^−^NK1.1^+^ NK cells in CD19^−^ mononuclear cells from different group of mice was explored by FACS. The results demonstrated that the percentage of NK cells from infected WT mice (0.80 ± 0.28%) was much less than that of uninfected WT mice (3.76 ± 0.56%, *P* < 0.01). So was the comparison between infected (1.33 ± 0.14%) and noninfected (3.43 ± 0.13%, *P* < 0.01) TLR3 KO mice. It was notable that the percentage of NK cells from TLR3 KO infected mice increased compared with WT infected mice (*P* < 0.01, Figure 1(b)).

Next, the expression of MHC II, CD69, NKG2A/C/E, and NKG2D on NK cells of mice from four groups was detected by using flow cytometry (Figure 1(c)). The expression of MHC II on NK cells from WT mice increased significantly after *S. japonicum* infection (6.15 ± 1.95% versus 16.36 ± 2.93%, *P* < 0.05, Figure 1(d)). MHC II expression on NK cells of TLR3 KO mice, by contrast, did not change after infection in statistics (5.94 ± 2.25% versus 5.97 ± 0.94%, *P* > 0.05, Figure 1(d)). The percentage of MHC II-expressing NK cells from TLR3 KO mice was less than WT after infection in statistics (*P* < 0.05, Figure 1(d)). Similar to MHC II, CD69 expression on NK cells from WT mice also increased significantly after infection (8.34 ± 2.18% versus 15.39 ± 0.69%, *P* < 0.05, Figure 1(d)), and there was no difference between infected and noninfected TLR3 KO mice (11.10 ± 2.04% versus 10.66 ± 3.37%, *P* > 0.05, Figure 1(d)). However, expression of CD69 on NK cells from infected TLR3 KO mice did not change obviously compared with their matched control group (*P* > 0.05, Figure 1(d)). Consistent with our previous study, the expression of NKG2A/C/E and NKG2D on NK cells from WT mice decreased significantly after *S. japonicum* infection (NKG2A/C/E: 47.88 ± 8.12% versus 19.93 ± 3.69%; NKG2D: 80.62 ± 2.21% versus 35.35 ± 3.65%, *P* < 0.05, Figure 1(d)). As we expected, there was no obvious change between infected and noninfected TLR3 KO mice (NKG2A/C/E: 45.25 ± 2.42% versus 42.35 ± 6.65%; NKG2D: 67.17 ± 1.62% versus 60.51 ± 13.67%, *P* > 0.05, Figure 1(d)). Notably, expression of NKG2A/C/E and NKG2D on NK cells from infected TLR3 KO mice was much higher when compared with infected WT mice (*P* < 0.05, Figure 1(d)).

We also measured cytokine production of NK cells from infected TLR3 KO mice. Figures 1(e) and 1(f) showed that the percentage of IFN-*γ*^+^ NK cells from WT mice decreased obviously after *S. japonicum* infection (noninfected: 15.19 ± 2.62%; infected: 4.03 ± 0.25%, *P* < 0.05, Figure 1(f)) as our previous study, and so was the TLR3 KO group (noninfected:14.73 ± 4.4%; infected: 5.94 ± 0.6%, *P* < 0.05, Figure 1(f)). The percentages of IL-4^+^ and IL-5^+^ NK cells in noninfected WT mice were 3.86 ± 0.26% and 0.76 ± 0.44%, respectively. These percentages increased significantly to 8.27 ± 0.70% (*P* < 0.05, Figure 1(f)) and 1.66 ± 0.22% (*P* < 0.05, Figure 1(f)), respectively, after *S. japonicum* infection. These numbers decreased significantly to 4.07 ± 1.77% and 0.70 ± 0.16% from infected TLR3 KO mice (*P* < 0.05, Figure 1(f)), which were equal to the noninfected TLR3 KO mice group (IL-4: 3.78 ± 0.09%; IL-5: 0.45 ± 0.13, *P* > 0.05, Figure 1(f)). Unexpectedly, the percentages of IL-17^+^ NK cells increased significantly after infection from both WT (noninfected: 1.74 ± 0.09%; infected: 5.06 ± 1.71%, *P* < 0.05, Figure 1(f)) and TLR3 KO mice (noninfected: 1.31 ± 0.05%; infected: 2.31 ± 0.38%, *P* < 0.05, Figure 1(f)). Still, there were significant differences between WT and TLR3 KO infected mice (*P* < 0.05, Figure 1(f)).

## 4. Discussion

It was reported that NK cells could express many kinds of TLRs, which could play an important role in the progress of NK cell activation in response to bacterial and viral infection [21] and tumors [24]. Here, roles of TLRs on NK cells in the progress of *S. japonicum* infection were investigated in the spleen of C57BL/6 mice after infection for 6 weeks, which is the acute phase of infection in previous report [6–8]. As shown in Figure 2, splenic NK cells expressed a higher level of TLR3 than TLR2, TLR4, TLR7, and TLR9 from *S. japonicum*-infected mouse (*P* < 0.05). It suggested that TLR3 might be involved in modulating the activation of *S. japonicum* infection-induced NK cell.

As we know that *S. japonicum* infection could induce a Th2-dominant immune response in the body [25]. Our previous research has found that the ability of splenic NK cells in secreting IFN-*γ* from *S. japonicum*-infected mouse is decreased while the IL-4 secretion is increased [7]. Here, our results demonstrated that Poly I:C could induce a higher level of IL-4 from infected mouse splenocytes (Figure 3(a), *P* < 0.05) and the percentage of IL-4^+^ NK cells in poly I:C cocultured infected splenocytes was extremely higher (*P* < 0.05). It suggested that *S. japonicum* infection-induced TLR3 on NK cells was involved in modulating the function of NK cells. However, it was reported that poly I:C is not only a TLR3 ligand but also can interact with RIG-I [26, 27], which might influence the expression of IL-4 [28].

On the other hand, it was reported that TLR3 could modulate immunopathology during *Schistosoma mansoni* egg-driven Th2 responses in the lung [19]. Many types of immune cells express TLR3, such as DC cells, macrophages, NK cells, T cells, and B cells [29–32]. In this study, the expression of TLR3 was examined on different types of splenocytes from normal and *S. japonicum*-infected mice. Results showed that TLR3 expression only increased obviously in NK cell from infected mice (*P* < 0.01, Figure 4(a)). It suggested that NK cells might be the main target cells which could respond to materials through TLR3 in the spleen in the course of *S. japonicum* infection. Although the engagement of both TLR2 and TLR3 by schistosome eggs is important for the production of inflammatory cytokines and interferon-stimulated genes, such as some chemokines, by DCs [18], no significant differences of TLR3 expressions were observed in pDCs, cDCs, neutrophils, and macrophages (*P* > 0.05, Figure 4(b)). It might relate to the fact that myeloid cells mainly play function on the site of local inflammation [33].

MHC II, CD69, NKG2A/C/E, and NKG2D are activation- and function-associated molecules which were expressed on the surface of NK cells [34–37]. Our results demonstrated that the percentages of MHC II-, CD69-, and NKG2A/C/E-expressing cells in TLR3^+^ NK cells increased significantly after infection (*P* < 0.05, Figures 5(a) and 5(b)). It indicated that *S. japonicum* infection could induce the activation of TLR3^+^ NK cell in the spleen. Moreover, it was reported that NK lineage may develop into cytokine-producing/APCs which affect the priming and progress of systemic autoimmune disease [37]. And many cytokines, such as IFN-*γ*, IL-4, IL-5, and IL-17, were reported to be secreted by NK cells [7, 38]. Our results showed a significant increase in the percentage of IL-4-, IL-5-, and IL-17-producing cells in TLR3^+^ NK cells population from infected mice (*P* < 0.05, Figures 5(c) and 5(d)). It indicated that TLR3 might mediate the Th2-like immune response of splenic NK cells in the progress of *S. japonicum* infection.

Additionally, TLR3^−/−^ mice were infected with *S. japonicum* to further evaluate the role of TLR3 on NK cells during infection. Results showed that the expression of NKG2A/C/E, NKG2D, MHC II, and CD69 on NK cells was decreased significantly in *S. japonicum*-infected TLR3^−/−^ mice (Figures 1(c) and 1(d)). These results suggested that TLR3 mediated the activation of NK cells during *S. japonicum* infection. Meanwhile, our results showed the significant decrease of IFN-*γ*-producing NK cells and increase of IL-4-, IL-5-, and IL-17-producing NK cells in *S. japonicum*-infected TLR3^−/−^ mice (*P* < 0.05, Figures 1(e) and 1(f)). It confirmed that TLR3 could modulate the function of NK cells in the course of *S. japonicum* infection.

In summary, our study demonstrated that splenic NK cells from *S. japonicum*-infected C57BL/6 mice expressed a higher level of TLR3, and TLR3-expressing NK cells were more sensitive, both in vitro and in vivo. Our results suggested that TLR3 might be involved in modulating the immune response of NK cells in the course of *S. japonicum* infection in C57BL/6 mice.

## Data Availability

All data used to support the findings of this study are available from the corresponding author upon request.

## Abbreviations

*S. japonicum*: *Schistosoma japonicum*
TLR: Toll-like receptor
NK: Nature killer
IL: Interleukin
Th: T helper cell
IFN: Interferon
KO: Knockout
PRRs: Pattern recognition receptors
PMA: Phorbol 12-myristate 13-acetate.
